# Intubation in Swine: What Recumbency to Choose?

**DOI:** 10.3390/ani12182430

**Published:** 2022-09-15

**Authors:** Alessandro Mirra, Claudia Spadavecchia, Fabiana Micieli

**Affiliations:** 1 Section of Anaesthesiology and Pain Therapy, Department of Clinical Veterinary Medicine, Vetsuisse Faculty, University of Bern, 3012 Bern, Switzerland; 2 Graduate School for Cellular and Biomedical Sciences, University of Bern, 3012 Bern, Switzerland; 3 Department of Veterinary Medicine and Animal Production, University of Naples “Federico II”, 80137 Naples, Italy

**Keywords:** pig, intubation, recumbency, veterinary, anaesthesia, sternal, dorsal

## Abstract

**Simple Summary:**

Whether a pig should be placed on its back or sternum to facilitate endotracheal intubation is currently not known and a subject of debate. Enrolling participants with different clinical anaesthesia backgrounds, we aimed at assessing which recumbency (dorsal versus sternal) is advantageous, and if operator experience can influence the results. Using objective and subjective outcomes, we found that sternal recumbency is advantageous if compared to dorsal, but that this is true only for operators lacking experience in anaesthetising animals. Experts’ confidence in intubating various animal species with different techniques probably minimized the influence of recumbency.

**Abstract:**

Endotracheal intubation (ETI) is challenging in pigs. We compared the number of attempts and time to perform ETI, and the subjective perception of ease, while the animal was positioned in dorsal (DR) or sternal (SR) recumbency, as well as assessed whether operator experience influences the outcome. Participants were divided into three groups: undergraduates (ST; veterinary students), graduates (GR; veterinarians without specific anaesthesia training) and experts (EX; veterinary anaesthesia intern/resident and diplomate of the European College of Veterinary Anaesthesia and Analgesia). Each participant intubated one freshly euthanised pig in DR and ST. Number of attempts and time to correctly perform ETI, number of oesophageal intubations and answers to Likert-scale questions on larynx visualization and ease of endotracheal tube introduction and advancement were recorded. Thirty-three participants were enrolled (15 ST, 10 GR and 8 EX). Less attempts (*p* = 0.002) and time (*p* = 0.002) to correctly perform ETI were needed in SR for the ST group. In 21/119 and 5/48 ETI attempts, oesophageal intubation was performed in DR and SR, respectively. Larynx visualization (*p* < 0.001) and endotracheal tube introduction (*p* < 0.001) were perceived as easier in SR for the ST group. No difference between recumbencies was found in perceived ease to advance the endotracheal tube. For inexperienced operators, intubation in SR can be recommended.

## 1. Introduction

Airway management of pigs undergoing general anaesthesia can be challenging [1,2,3]. While for long procedures intubation is widely recommended to reduce morbidity and mortality [4], the long snout, narrow oropharyngeal cavity, thick tongue, elongated soft palate and curved larynx represent substantial obstacles to its performance [1]. Moreover, a tracheal bronchus, ventilating an accessory right lobe, is present in pigs. Even if it does not constitute an obstacle for the endotracheal intubation per se, it can be bypassed or intubated, adding a further challenge to the airway management in this species [1,5]. Despite the high number of pigs undergoing experimental surgery every year [6], little information is available regarding the most advantageous animal positioning to perform intubation. Indeed, recommendations are mostly based on personal experience [4,7]. One clinical study reported a shorter intubation time with the pigs in sternal (SR) compared to dorsal (DR) recumbency [2]; however, only a single investigator performed all the procedures. The aims of this study were to (1) compare the number of attempts and the time needed to perform a correct endotracheal intubation (ETI); (2) compare the operator perception of ease to visualize the larynx, to introduce the endotracheal tube (ETT) and to advance the ETT, with the animal positioned in DR versus SR; and (3) to assess whether operator experience can influence the outcomes. We hypothesized that DR will allow a better alignment of the upper airway anatomical structures, thus leading to a faster intubation with less attempts for all participants. However, we also hypothesised that previous experience in anaesthesia with other animal species will facilitate the intubation in SR.

## 2. Materials and Methods

Participants were enrolled from the Veterinary Faculty and the Experimental Surgery Facility of the University of Bern. Three groups were foreseen: undergraduates (ST; veterinary students), graduates (GR; veterinarians without specific training in anaesthesia) and experts (EX; veterinary anaesthesia intern/resident and diplomate of the European College of Veterinary Anaesthesia and Analgesia).

An invitation to participate in the study was sent to students (ST group) via email. Participants belonging to the GR and EX groups were personally invited. No ethical authorization was needed for the present study according to the judgement of the competent authorities (Business Administration System for Ethics Committees, number Req-2021-00286).

Sixteen juvenile mixed sex pigs, freshly euthanized for a separate experimental trial, were used for the present study. The animals’ age and weight (mean (standard deviation)) were 10.8 (0.6) weeks and 28.9 (4.7) kg, respectively. In order to avoid bias due to deformation of the upper airway tissues caused by repeated intubations, a maximum of three participants per pig was set. Thus, a maximum of 48 participants was foreseen.

Within the 48 h preceding the trial, each participant (except for experts) received a file explaining how to perform pig intubation in both recumbencies. The investigation was always performed in the early afternoon, within one hour after euthanasia. Each participant intubated the pig in DR and SR, following a computer-generated random order (https://www.randomizer.org/, (accessed in January 2021). In case of intubation in DR, no help was provided; in case of intubation in ST, one investigator (AM) assisted with mouth opening and moving the animal’s head, as requested by the participant. Number of attempts and time to perform a correct endotracheal intubation (ETI) were recorded. The timing of the procedure began when the laryngoscope was placed on the tongue and ended when a correct ETI was confirmed by one of the main investigators (FM; the mouth was opened and endotracheal positioning of the tube visually confirmed). Intubation time was calculated summing the time of each single attempt (i.e., pauses between attempts were not taken in consideration). The number of oesophageal intubations was recorded and the soft palate entrapment (presence/absence) was evaluated, by one of the main investigators (FM or AM), during the first attempt, for each recumbency and participant.

After the procedure, an online questionnaire consisting of three Likert-scale questions was filled-up by each participant. Respondents indicated their agreement with the statement: strongly disagree; disagree; neither agree nor disagree; agree; strongly agree. Questions were structured as clear, concise and fulfilling the end goal, avoiding assumptions:

(1) I can easily visualize the larynx;
(2) I can easily introduce the endotracheal tube;
(3) I can easily advance the endotracheal tube.

Finally, one open-ended question was also asked: “Which technique (dorsal or sternal recumbency) would you choose to intubate a pig? Explain your preference”.

Statistical analysis was performed using the software SigmaPlot Version 14.0 (Systat Software Inc., San Jose, CA, USA). Normality was assessed using the Shapiro–Wilk test. Data with a normal distribution are presented as the mean (standard deviation) and those with a non-normal distribution as the median (interquartile ranges). For all the Likert-scale questions, the possible answer options were scored into five progressive point categories (strongly disagree = 1; disagree = 2; neither agree nor disagree = 3; agree = 4; strongly agree = 5). In order to evaluate the difference in answers to the Likert-scale questions, the number of attempts and total intubation time between DR and SR, the Wilcoxon Signed Rank Test was used. Comparison among groups was performed using the Kruskal–Wallis one-way analysis of variance on ranks, followed by Dunn’s Method, when appropriate. Level of significance was defined as *p* < 0.05. Answers to the open-ended question were classified according to the items reported.

## 3. Results

Thirty-three participants were enrolled in the study (15 ST, 10 GR and 8 EX). Among the students, none had previously performed an intubation.

**Figure 1:**
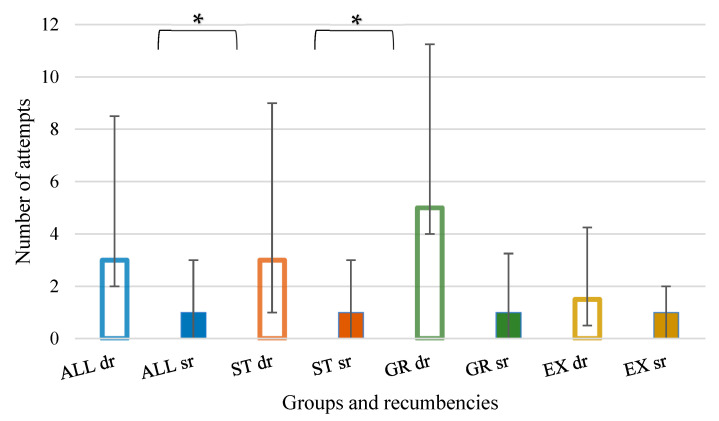
Number of attempts needed to achieve a correct endotracheal intubation. Groups are identified by colours: blue—all; orange—undergraduates (ST); green—graduates (GR); yellow—experts (EX). Recumbencies are identified by bar fill: dorsal recumbency (dr)—no fill; sternal recumbency (sr)—full fill. * Statistically significant difference between recumbencies.

Less attempts (*p* < 0.001) were needed to perform a correct ETI in SR compared to DR (Figure 1).

**Figure 2:**
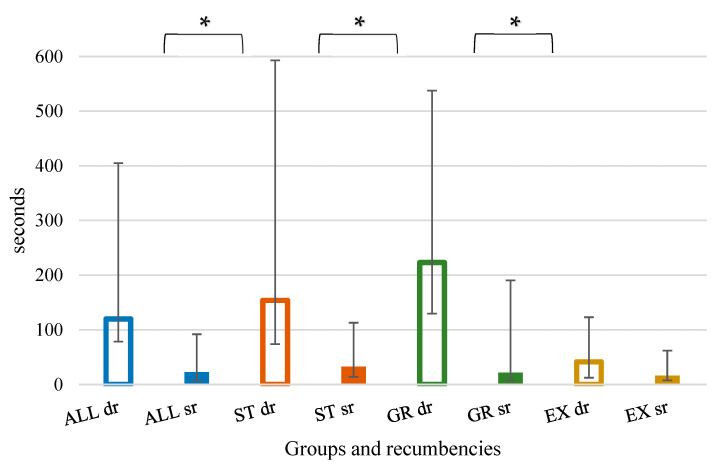
Time (seconds) needed to achieve a correct endotracheal intubation. Groups are identified by colours: blue—all; orange—undergraduates (ST); green—graduates (GR); yellow—experts (EX). Recumbencies are identified by bar fill: dorsal recumbency (dr)—no fill; sternal recumbency (sr)—full fill. * Statistically significant difference between recumbencies.

Similarly, less time (*p* < 0.001) was needed to perform a correct ETI in SR compared to DR (Figure 2).

Out of a total of 119 and 48 intubation attempts in DR and SR, respectively, 21 (17.6%) and 5 (10.4%) times an oesophageal intubation was performed. Analysing the answers to the questionnaire, larynx visualization (*p* < 0.001) and endotracheal tube introduction (*p* < 0.001) were judged to be easier in SR. No difference was found between recumbencies in the ease of advancing the endotracheal tube (Table 1).

Occurrence of soft palate entrapments is reported in Table 2.

### Subgroup Analysis

Less attempts (*p* = 0.002) to correctly perform ETI were needed in SR for the ST group, but not for the GR and EX groups (Figure 1 and Figure 2). Similarly, less time to perform it was needed in SR for the ST group (*p* = 0.002) and the GR group (*p* = 0.037), but not for the EX group.

Analysing the answers to the questionnaire, larynx visualization (*p* < 0.001) and endotracheal tube introduction (*p* < 0.001) were easier in SR for the ST group, but not for the GR and EX groups. No significant difference between recumbencies was found in the ease to advance the endotracheal tube in all groups (Table 1).

In the majority of the cases, SR was preferred to DR (24/33 participants: ST 11/15; GR 9/10; EX 4/8). Two participants in the ST group and one in the EX group did not express any preference. Mentioned reasons for recumbency preference (answer to open ended question) were visualization, second person help, ease of ETT advancement, laryngoscope ergonomic holding, landmarks recognition and previous experience. The main reasons were visualization (16/24) and previous experience (5/13; students not included due to lack of previous experience).

When differences among groups were assessed, ETT introduction in DR was deemed significantly easier for the EX group compared to the ST group (*p* = 0.01), and the time to perform it was significantly lower for the EX group compared to the GR group (*p* = 0.048). No other differences among groups were detected.

## 4. Discussion

In the present study, overall, fewer attempts and faster intubation were achieved with the animal in SR, which was also correlated with fewer oesophageal intubations. Moreover, larynx visualization and ETT introduction were perceived to be easier in SR compared to DR.

However, to notice is that most of the statistical differences decreased or disappeared with increasing operator experience. Indeed, while the ST group mirrored the overall results, only the time to perform a correct ETI was statistically less in SR for the GR group, and no differences between recumbencies were found in any of the considered outcomes for the EX group.

Initially, we hypothesized that the higher number of small-animal anaesthesia performed at our faculty would have created a bias in the more experienced participants in favour of SR, most commonly adopted for dogs and cats. However, the results from the present study show that the wider the anaesthesia experience, the lower the preference toward one of the two approaches. Confidence in intubating various animal species with different techniques could have minimized the influence of recumbency on the outcomes.

Ease of larynx visualization was shown to be a critical issue during intubation in DR. The veterinary literature typically provides a description of anatomical structures with the animals in a standing position; this could have facilitated the recognition of the anatomical landmarks with the animal in SR. The higher number of oesophageal intubations performed by students with the animal positioned in DR supports this theory.

The outcomes of the present study are in line with those of Theisen et al. [2], who recommended the use of the SR for intubating pigs. However, in that study, only intubation time was assessed (58.4 (47.4, 118.0) seconds in DR versus 17.3 (13.6, 29.9) seconds in SR) and intubation was always performed by the same operator. In the present study, a similar intubation time was found (120 (41.5, 285) seconds in DR versus 23 (15.5, 69) seconds in SR). As expected, the time needed diminished with increasing operator experience. This is in accordance with the results of Langton et al. in dogs [8], who reported a longer intubation time needed by students (54.2 (31.3) s) compared to veterinary surgeons (11.7 (8.5) s).

Soft palate entrapment more often was found with the animal in DR compared to SR. We cannot infer that the occurrence would be higher in live animals, but the higher prevalence of this observation in DR could have prolonged the intubation time, increased the number of attempts and made the overall intubation performance more difficult.

The present study has some limitations. First, only juvenile pigs were included. Even if animals around this age are commonly used for experimental studies, care should be taken in translating these results to adult animals. Second, participants in the different groups were not homogeneous due to the voluntary-based enrolment. Third, participants knew they were being observed, possibly leading to increased stress and anxiety, perhaps influencing their performance. However, each participant accomplished intubation in both recumbencies; thus, the preference toward one or the other should not have been influenced.

## 5. Conclusions

Intubation in SR seems to be advantageous, but only for inexperienced operators. Experts’ confidence in intubating various animal species with different techniques probably minimized the influence of recumbency on the outcomes.

## Figures and Tables

**Table 1:** Answers to the three Likert questions. The subgroup analysis and the number of participants for each group are also reported.

Question		Group	Recumbency		Percentage of Answer				Median Value (Q1, Q3)	*p*	Number of Participants
				Strongly Disagree	Disagree	Neither Agree Nor Disagree	Agree	Strongly Agree			
Q1	l can easily visualize the larynx	All	Dorsal	3%	36%	21%	24%	24%	3 (2, 4)	<0.001	33
			Sternal	0%	3%	3%	24%	24%	5 (4, 5)		
Q2	l can easily introduce the endotracheal tube	All	Dorsal	0%	36%	24%	21%	21%	3 (2, 4)	0.012	33
			Sternal	0%	15%	6%	42%	42%	4 (4, 5)		
Q3	l can easily advance the endotracheal tube	All	Dorsal	0%	21%	15%	42%	42%	4 (3, 4)	0.969	33
			Sternal	0%	24%	15%	33%	33%	4 (2.5, 5)		
Q1	l can easily visualize the larynx	ST	Dorsal	7%	47%	20%	20%	20%	2 (2, 4)	<0.001	15
			Sternal	0%	0%	7%	13%	13%	5 (5, 5)		
		GR	Dorsal	0%	30%	30%	30%	30%	3 (2, 4)	0.098	10
			Sternal	0%	10%	0%	30%	30%	5 (4, 5)		
		EX	Dorsal	0%	25%	13%	25%	25%	4 (2.25, 5)	0.219	8
			Sternal	0%	0%	0%	38%	38%	5 (4, 5)		
Q2	l can easily introduce the endotracheal tube	ST	Dorsal	0%	47%	33%	20%	20%	3 (2, 3)	<0.001	15
			Sternal	0%	7%	7%	40%	40%	4 (4, 5)		
		GR	Dorsal	0%	40%	20%	40%	40%	3 (2, 4)	0.313	10
			Sternal	0%	30%	0%	40%	40%	4 (2, 5)		
		EX	Dorsal	0%	13%	13%	0%	0%	5 (3.5, 5)	0.438	8
			Sternal	0%	13%	13%	50%	50%	4 (3.25, 4.75)		
Q3	l can easily advance the endotracheal tube	ST	Dorsal	0%	20%	20%	47%	47%	4 (3, 4)	0.275	15
			Sternal	0%	7%	20%	47%	47%	4 (3, 5)		
		GR	Dorsal	0%	30%	20%	40%	40%	3.5 (2, 4)	0.742	10
			Sternal	0%	40%	0%	20%	20%	4 (2, 5)		
		EX	Dorsal	0%	13%	0%	38%	38%	4.5 (4, 5)	0.109	8
			Sternal	0%	38%	25%	25%	25%	3 (2, 4)		

**Table 2:** Occurrence of soft palate entrapments. The subgroup analysis is also reported.

Group	Recumbency	Event/Number of Participants
All	Dorsal	11/33
	Ventral	5/33
ST	Dorsal	6/15
	Ventral	3/15
GR	Dorsal	4/10
	Ventral	2/10
EX	Dorsal	1/8
	Ventral	0/8

## Data Availability

Data is contained within the article.

## References

[1] Moon P.F., Smith L.J. (1996). General anesthetic techniques in swine. Vet. Clin. N. Am. Food Anim. Pract..

[2] Theisen M.M., Maas M., Hartlage M.A.G., Ploner F., Niehues S.M., Van Aken H.K., Weber T.P., Unger J.K. (2009). Ventral recumbency is crucial for fast and safe orotracheal intubation in laboratory swine. Lab. Anim..

[3] Anderson D.E., Mulon P.Y. (2019). Diseases of Swine. Diseases of Swine.

[4] Kaiser G.M., Heuer M.M., Frühauf N.R., Kühne C.A., Broelsch C.E. (2006). General handling and anesthesia for experimental surgery in pigs. J. Surg. Res..

[5] Tonge M., Robson K. (2021). Hypoxaemia following suspected intubation of the tracheal bronchus of a pig. Vet. Rec. Case Rep..

[6] European Commission (2020). European Commission Report from the Commission to the European Parliament and the Council 2019: Report on the Statistics on the Use of Animals for Scientific Purposes in the Member States of the European Union in 2015–2017.

[7] Schmitz T., Konrad R.M., Tarbiat S. (1964). Zur Technik der Intubationsnarkose bei Läuferschweinen. Z. Gesamte Exp. Med..

[8] Langton S.D., Blevins M.B. (2021). The time required to achieve endotracheal intubation in dogs: A comparison of veterinary students and qualified veterinary surgeons. Vet. Anaesth. Analg..

